# Estimating the Proportion of Transplant-Eligible Patients in Diffuse Large B-Cell Lymphoma: Insights from German Hospital Billing Data

**DOI:** 10.1159/ort/aecag003

**Published:** 2026-08-31

**Authors:** Ann-Cathrine Froitzheim, Melina Sophie Kurte, Tabea Poos, Udo Holtick, Florian Kron

**Affiliations:** a VITIS Healthcare Group, Cologne, Germany; b Faculty of Medicine, University of Duisburg-Essen, Essen, Germany; c Department I of Internal Medicine, Center for Integrated Oncology (CIO Cologne), Faculty of Medicine and University Hospital Cologne, University of Cologne, Cologne, Germany; d FOM University of Applied Sciences, Essen, Germany

**Keywords:** Diffuse large B-cell lymphoma, SCT eligibility, Transplant eligibility, High-dose chemotherapy and autologous stem cell transplantation, Reimbursement, Inpatient billing data

## Abstract

**Introduction:**

Treatment, according to guidelines, for second-line and beyond (2L+) therapy of diffuse large B-cell lymphoma (DLBCL) differentiates between transplant-eligible and transplant-ineligible patients. Transplant-eligible patients typically receive salvage immunochemotherapy followed by high-dose chemotherapy and autologous stem cell transplantation (HD-ASCT) or chimeric antigen receptor T-cell therapy. Although literature suggests a 50-50 distribution between transplant-eligible and transplant-ineligible patients, real-world evidence from Germany is lacking. This study aimed to estimate the proportion of transplant-eligible and transplant-ineligible patients using intended-for-HD-ASCT national inpatient billing data to evaluate the validity of the commonly assumed distribution.

**Methods:**

Inpatient billing data from all German hospitals in 2024, provided by the Institute for the Hospital Remuneration System (InEK), were analyzed. Cases with the main diagnosis “C83.3” (DLBCL) defined the base population. Applying guideline-based first-line cure rates (60% [minimum]–70% [maximum]), the 2L population size was estimated. Transplant-eligible cases were identified via operation and procedure score codes for salvage immunochemotherapies (R-ICE, R-DHAP, R-GDP). The transplant-eligible proportion was calculated by dividing the number of transplant-eligible cases by the estimated 2L population. Sensitivity analyses were performed using data from 2022 to 2023, validating the 2L relapse/refractoriness rate assumption against German real-world claims data, and assessing potential impact of polatuzumab vedotin-related coding bias.

**Results:**

The base population included 32,851 DLBCL cases. Based on cure rate assumptions, the estimated 2L population size ranged from 9,855 (minimum) to 13,140 (maximum). A total of 7,523 cases were identified to have received salvage immunochemotherapy based on coding criteria. This corresponds to a transplant-eligible proportion of patients intended for HD-ASCT of 57% (minimum) and 76% (maximum). Sensitivity analyses yielded a transplant-eligible proportion range of 40–54% (2022) and 52–69% (2023), 52% when applying the relapse/refractoriness rate from German real-world claims data, and 34–46% when excluding polatuzumab vedotin-related cases.

**Conclusion:**

This analysis introduces a method for approximating the distribution of transplant eligibility among DLBCL patients intended for HD-ASCT in the 2L setting using real-world inpatient billing data. The results question the widely assumed balance between transplant-eligible and transplant-ineligible groups, indicating a possible tendency toward a slightly higher proportion of transplant-eligible cases which may be clinically eligible for HD-ASCT.

## Introduction

The therapy landscape for patients with diffuse large B-cell lymphoma (DLBCL), the most common subtype of non-Hodgkin lymphoma, has evolved significantly over the past few years [1, 2]. In recent years, the combination of chemotherapy and immunotherapy with the anti-CD20 monoclonal antibody rituximab has led to a significant reduction in the incidence of primary treatment failure and disease relapse in patients with DLBCL [3]. Hence, the combination of rituximab and chemotherapy R-CHOP (cyclophosphamide, doxorubicin, vincristine, and prednisone) remains the predominant first-line treatment [4]. Recently, Pola-R-CHP has also been approved for use in the first-line setting [5, 6]. However, approximately 30–40% of patients require treatment for either relapsed or primary refractory DLBCL [7, 8]. Decisions for second-line therapy and beyond are individualized and based on prior disease course, previous treatments, patient age, and comorbidities, taking into account the specific risk-benefit profile [9]. Possible treatment alternatives include autologous stem-cell transplantation, chimeric antigen receptor (CAR) T-cell therapy, conventional chemotherapy, and immunotherapy [10]. To approach the therapy decision, patients will be assessed to determine whether they are eligible for high-dose chemotherapy and subsequent autologous stem cell transplantation (HD-ASCT) or not [11]. Intended transplant eligibility may diverge from clinically confirmed eligibility, as the latter additionally depends on adequate response to preceding salvage immunochemotherapy [7]. If not transplant-eligible, patients are recommended to receive conventional or targeted therapies [7]. Although categorizing patients as transplant-eligible or transplant-ineligible is no longer fully adequate in light of the broad availability of second-line therapies, especially CAR-T therapies and bi-specific antibodies treatment regimens, this classification continues to be used in German health technology assessment approaches [7, 8, 12, 13]. Furthermore, the latest developments of guideline recommendations indicate CAR-T therapy as an additional alternative to managing transplant-eligible patients apart from HD-ASCT, e.g., primary-refractory or early relapse patients [7, 8, 14, 15].

Randomized trial data from 2003 to 2008 (CORAL trial, NCT00137995) suggests an approximate allocation of transplant-eligible and transplant-ineligible patients in DLBCL of 50% each [11, 16]. Ever since 2008, the therapy landscape has substantially evolved. Thus, recent validated real-world data, relevant, for example, to health services research, are limited. German hospital billing data offers an approach to address this gap, as coding of diagnoses and performed procedures allows drawing conclusions on performed therapy details on an aggregated level. Given these prementioned developments regarding the actual clinical eligibility for and the use of HD-ASCT, this study aims to verify the 50-50 distribution of the transplant-eligible vs. transplant-ineligible DLBCL patients intended for HD-ASCT size given by the literature and approximate corresponding quantities based on real-world data.

## Methods

### Data Base and Underlying Assumption of the Analysis

The Data Browser of the German Institute for the Hospital Remuneration System (InEK) was used as underlying source of this analysis [17]. The InEK Data Browser provides publicly available, real-world inpatient billing data submitted by all German hospitals, containing anonymized, aggregated case-level data structured according to the German Diagnosis-Related Groups (DRG) system. The Browser allows filtering provided data by different characteristics such as patient age group, diagnoses (based on ICD-10-GM), performed procedures (based on operation and procedure [OPS] codes), or medical department. For this analysis, data were analyzed under the core assumption that receipt of salvage chemotherapy, which is indicated by certain OPS-codes in hospital billing data, serves a joint proxy for intended and clinically confirmed transplant eligibility (intended for HD-ASCT).

### Determination of Baseline Population

The analysis approach is depicted in Figure 1. The baseline population was defined as patients diagnosed with relapsed or refractory DLBCL (ICD-10: C83.3) entering the second-line treatment (2L) setting. The base year for the analysis was set for 2024, the latest full data year available at the time of the analysis. For estimation of the baseline population, the total number of 2024 hospital inpatient DLBCL cases (N_2024_) was retrieved from the Data Browser and then limited to the proportion of patients facing a relapsed or refractory form of the disease, which is between 30 and 40% according to the German guideline on DLBCL [7]. Consequently, two scenarios were generated comprising the maximum and the minimum base case population. Calculations were conducted according to:

$$\text{Base}\;{\text{population}}_{\min}=30\%\;x\;N_{2024},$$

$$\text{Base}\;{\text{population}}_{\max}=40\%\;x\;N_{2024}.$$

**Fig. 1.**
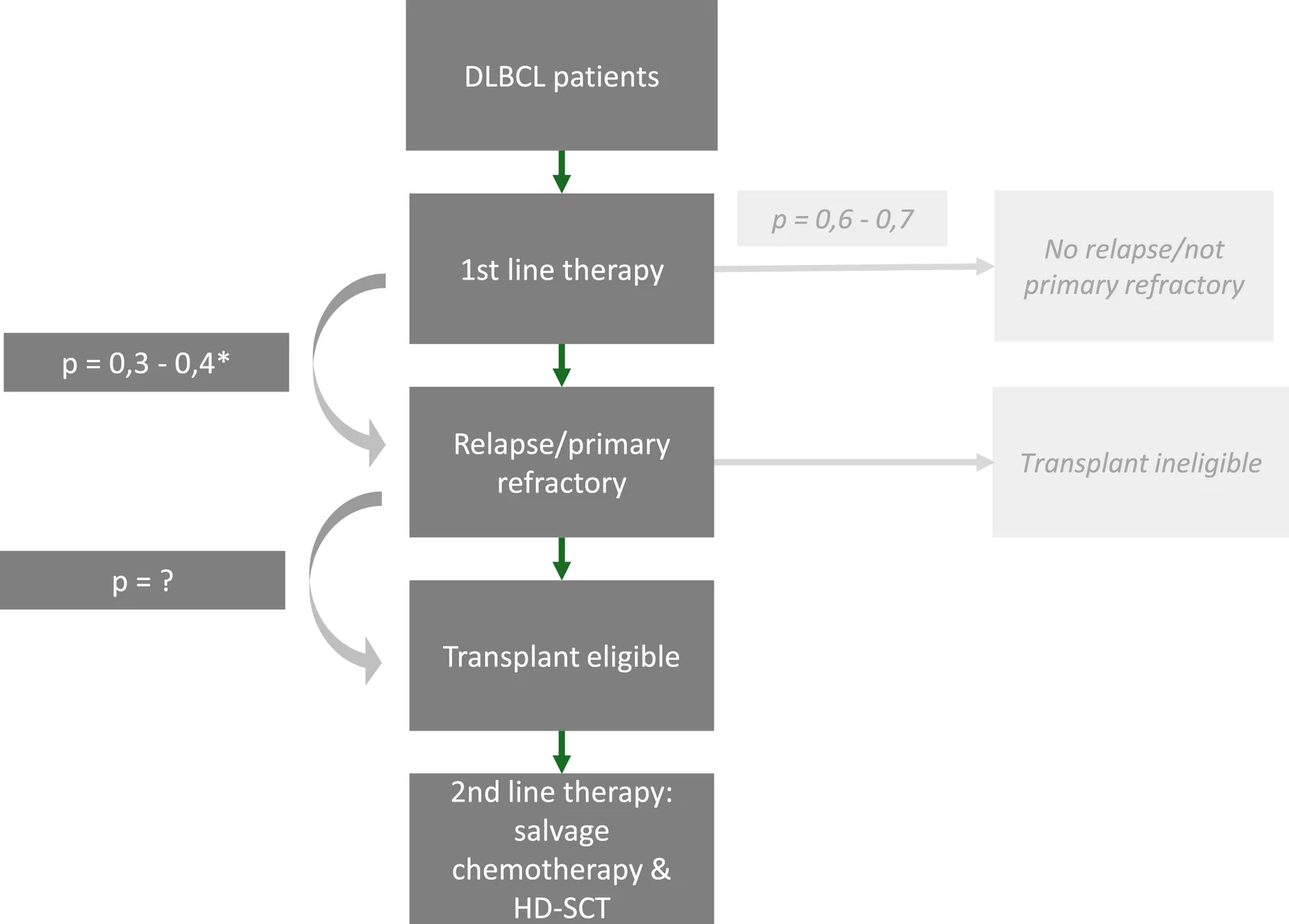
Analysis approach (*according to S3-Guideline [6]).

### Data Delimitation to Transplant Eligible Cases

The target population was defined as DLBCL inpatient cases in 2024 who received salvage chemotherapy, used as an indicator of subsequent HD-ASCT in the 2L setting. To identify these cases within the hospital billing data and narrow the C83.3 population down accordingly, clinical guidelines were screened to determine corresponding salvage chemotherapies. Afterward, the OPS-codes that reflect each chemotherapy were identified.

In the German DRG system, immunochemotherapy is coded through two components: an immunotherapy and a chemotherapy code. Where applicable, coding is supplemented by a drug-specific code that can trigger additional reimbursement. To capture immunotherapy, one OPS-code is required in all chemotherapy regimens (primary procedure code):

- 8-547.0 for “Other immunotherapy: with non-modified antibodies.”

The above-mentioned code is then supplemented with additional OPS according to the number of substances and the number of application days of the respective chemotherapy regimens (secondary procedure codes):

- 8-543.22 (“Moderately complex and intensive block chemotherapy: 2 days, 2 substances”)
- 8-544 (“Highly complex and intensive block chemotherapy”)
- 8-542.12 (“Not complex chemotherapy: 1 day, 2 substances”)
- 8-542.11 (“Not complex chemotherapy: 1 day, 1 substance”).

An overview of salvage chemotherapy regimens, corresponding substances, and OPS-codes is given in Table 1. Subsequently, the search strategy was constructed as follows (linked via the Boolean operator “AND”):

1. The main diagnosis (DLBCL, ICD-10 C83.3),
2. The primary procedure code (necessary condition),
3. Secondary procedure codes (sufficient condition, linked via Boolean operator “OR”).

**Table 1.** Salvage chemotherapies, substances, OPS-codes with explanation

Chemotherapy	Substances	Coding (OPS)
R-DHAP	R: rituximab	8-547.0 (Other immunotherapy: with non-modified antibodies)
	D: dexamethasone	8-543.22 (Moderately complex and intensive block chemotherapy: 2 days, 2 substances)
	HA: high-dose cytarabine	6-001.h/6-001.j (Administration of rituximab)
	P: cisplatin	​
R-ICE	R: rituximab	8-547.0 (Other immunotherapy: with non-modified antibodies)
	I: ifosfamide	8-544 (Highly complex and intensive block chemotherapy)
	C: carboplatin	6-001.h/6-001.j (Administration of rituximab)
	E: etoposide	​
R-GDP	R: rituximab	8-547.0 (Other immunotherapy: with non-modified antibodies)
	G: gemcitabine	8-542.12 (Not complex chemotherapy: 1 day, 2 substances)
	D: dexamethasone	8-542.11 (Not complex chemotherapy: 1 day, 1 substance)
	P: cisplatin	8-544 (Highly complex and intensive block chemotherapy)
		6-001.h/6-001.j (Administration of rituximab)

OPS, operation and procedure.

Due to the technical limitations of the search function that restricts the search for procedures via “AND” to more than two codes, the drug-specific code for rituximab administration (procedure codes 6-001.h/6-001.j) remained disregarded. However, the remaining codes sufficiently define the entire chemotherapy regime, including the addition of rituximab. An illustration of the search strategy is presented in Figure 2.

**Fig. 2.**
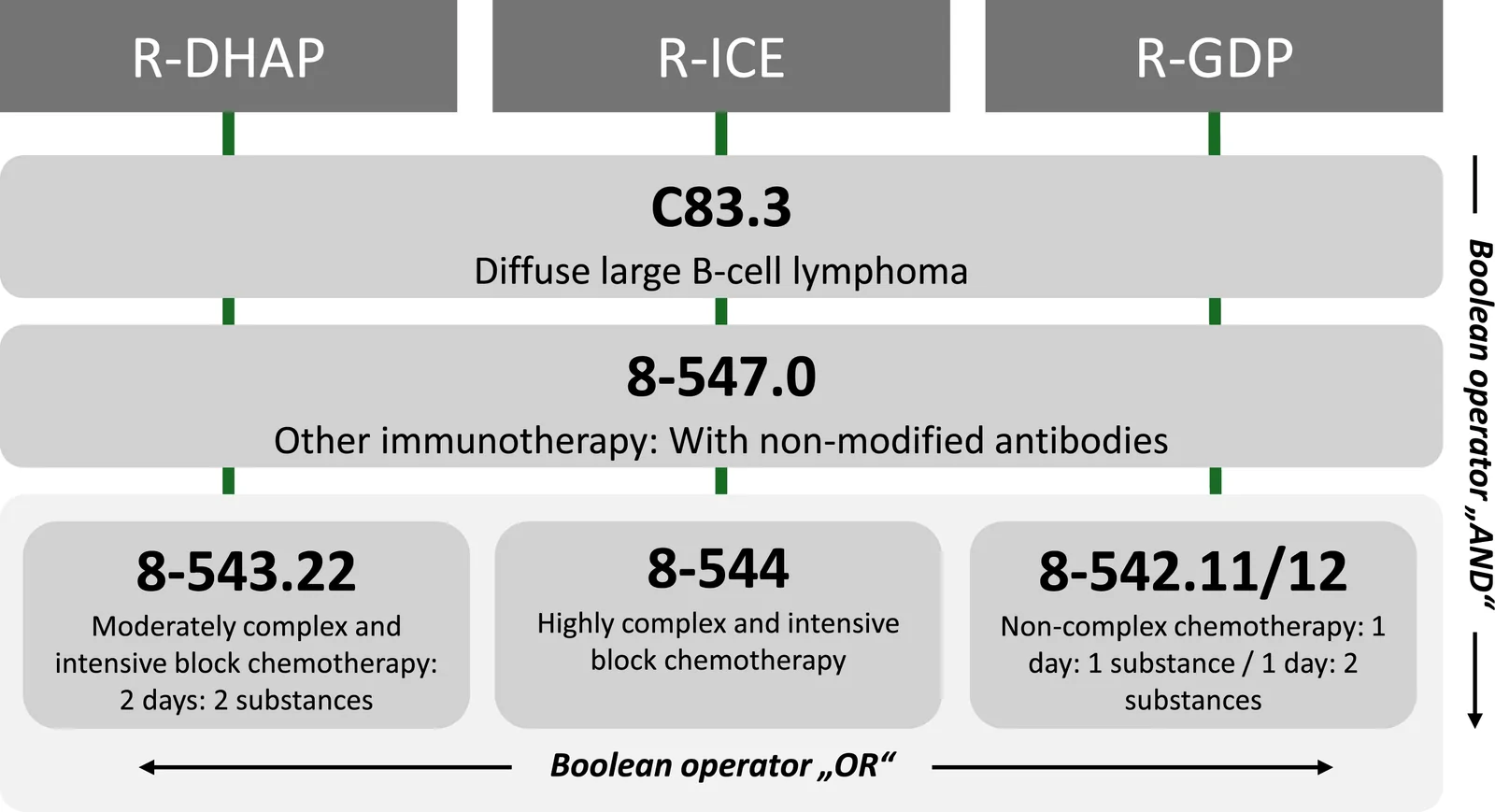
Search strategy for DLBCL salvage chemotherapy cases via InEK Data Browser.

### Approximating the Proportion of Transplant-Eligible Patients

Once the search strategy was applied, the number of cases of the secondary procedure codes (8-543.22, 8-544, 8-542.11/22) were added up. The resulting sum was used to calculate the relative minimum and maximum proportion of the target population of transplant-eligible r/r DLBCL patients according to the following equations:

Targetpopulationmin=∑n/8−543.22,8−544,8−542.1112÷Basepopulationmax

Targetpopulationmax=∑n/8−543.22,8−544,8−542.1112÷Basepopulationmin

### Sensitivity Analyses

To address uncertainty, three sensitivity analyses were performed. First, data were also analyzed for the years 2022 and 2023 to determine if the approach was robust, if data were consistent between the given years, or if trend deviations and fluctuation of case numbers over time were detectable. The data analysis procedure remained unchanged.

Second, to validate the main assumption for the 2L DLBCL population using a relapse/refractoriness rate of 30–40%, results from a large German real-world study were considered, drawing on health insurance claims data of patients newly diagnosed with DLBCL from 2010 to 2019. The findings show a broader population of 44% for 2L treatment, following completion of first-line therapy [18].

Third, the potential for bias was evaluated, as the Pola-BR regimen has increasingly been used in the second-line treatment setting for DLBCL following its approval in 2020 [6, 19]. Since the Pola-BR regimen is coded similarly to R-GDP, patients with a drug-specific OPS code for polatuzumab vedotin were identified and excluded from the analysis [20]. The proportion of transplant-eligible patients was subsequently approximated using the remaining patient population.

### Methodology Validation

The methodological approach was co-developed and validated by respective experts (hospital physician/oncologist, medical coder, medical controller, health economist).

## Results

### Baseline Population

InEK-data indicated 32,851 inpatient DLBCL cases for the year 2024. Under the assumption of 30–40% of patients receiving 2L treatment after relapse, the number of inpatient cases was 9,855 for base population_min_ and 13,140 for base population_max_.

### Transplant Eligible Population

Inpatient cases after narrowing down the 2024 DLBCL cases to those with specific coding for chemotherapy regimens R-DHAP, R-ICE, and R-GDP are presented in Table 2. Based on the common requirement (primary procedure code) that cases are coded as “Other immunotherapy: with non-modified antibodies”, a total of 7,523 cases were identified that were at the same time coded with either one of the secondary codes (8-544, 8-543.22, 8.542.12, 8-542.11). For the minimum and maximum target population size, 7,523 cases were divided firstly by 13,140 cases (base population_max_) and afterward by 9,855 cases (base population_min_), yielding a proportion range of 57% (target population_min_) to 76% (target population_max_) of cases classified as intended transplant-eligible.

**Table 2.** Summary of results of determining transplant-eligible cases for 2024

Inpatient cases DLBCL	r/r DLBCL cases (base population)	Regimen OPS codes	Number of cases	Sum of cases	Target population
32,851	min = 9,855	8-547.0 + **8-544**	2,489	7,523	min = 57.25%
	max = 13,140	8-547.0 + **8-543.22**	522		max = 76.34%
		8-547.0 + **8-542.12**	3,662		
		8-547.0 + **8-542.11**	850		

Data retrieved from InEK-Data Browser.

DLBCL, diffuse large B-cell lymphoma; OPS, operation and procedure.

### Sensitivity Analyses

For 2022, total inpatient DLBCL cases amounted to 31,668, with a range of 9,500 cases (base population_min_) to 12,667 cases (base population_max_) of relapsed or refractory DLBCL cases considering a relapse rate of 30–40%. Adding all cases coded with the primary code (Other immunotherapy: with non-modified antibodies, 8-547.0) and one of the secondary codes (8-544, 8-543.22, 8.542.12, 8-542.11), a total of 5,118 cases were reported. Calculations of the proportion of transplant-eligible cases ranged between 40% (5,118/12,667, target population_min_) and 54% (5,118/9,500, target population_max_). For 2023, 31,678 inpatient DLBCL cases were identified, resulting in 9,503 (min) to 12,671 (max) cases that are relapsed or refractory. DLBCL cases that were coded with the primary code and one of the secondary codes for considered chemotherapies added up to 6,580 inpatient cases. Subsequently, the proportion of transplant-eligible cases with a range of 52% (target population_min_) to 69% (target population_max_) was calculated. Table 3 shows an overview of data evaluated for the years 2022 and 2023. Figure 3 shows the development of estimated transplant-eligible case proportions over time, including results for 2024 (section 3.2).

**Table 3.** Summary of sensitivity analyses of determining transplant-eligible cases 2022 and 2023 retrieved from InEK-Data Browser

Year	Inpatient cases DLBCL	r/r DLBCL cases (base population)	Regimen OPS codes	Number of cases	Sum of cases	Target population
2022	31,668	min = 9,500	8-547.0 + **8-544**	2,184	5,118	min = 40.4%
		max = 12,667	8-547.0 + **8-543.22**	574		max = 53.87%
			8-547.0 + **8-542.12**	1,620		
			8-547.0 + **8-542.11**	740		
2023	31,678	min = 9,503	8-547.0 + **8-544**	2,340	6,580	min = 51.93%
		max = 12,671	8-547.0 + **8-543.22**	506		max = 69.24%
			8-547.0 + **8-542.12**	2,923		
			8-547.0 + **8-542.11**	811		

DLBCL, diffuse large B-cell lymphoma; OPS, operation and procedure.

**Fig. 3.**
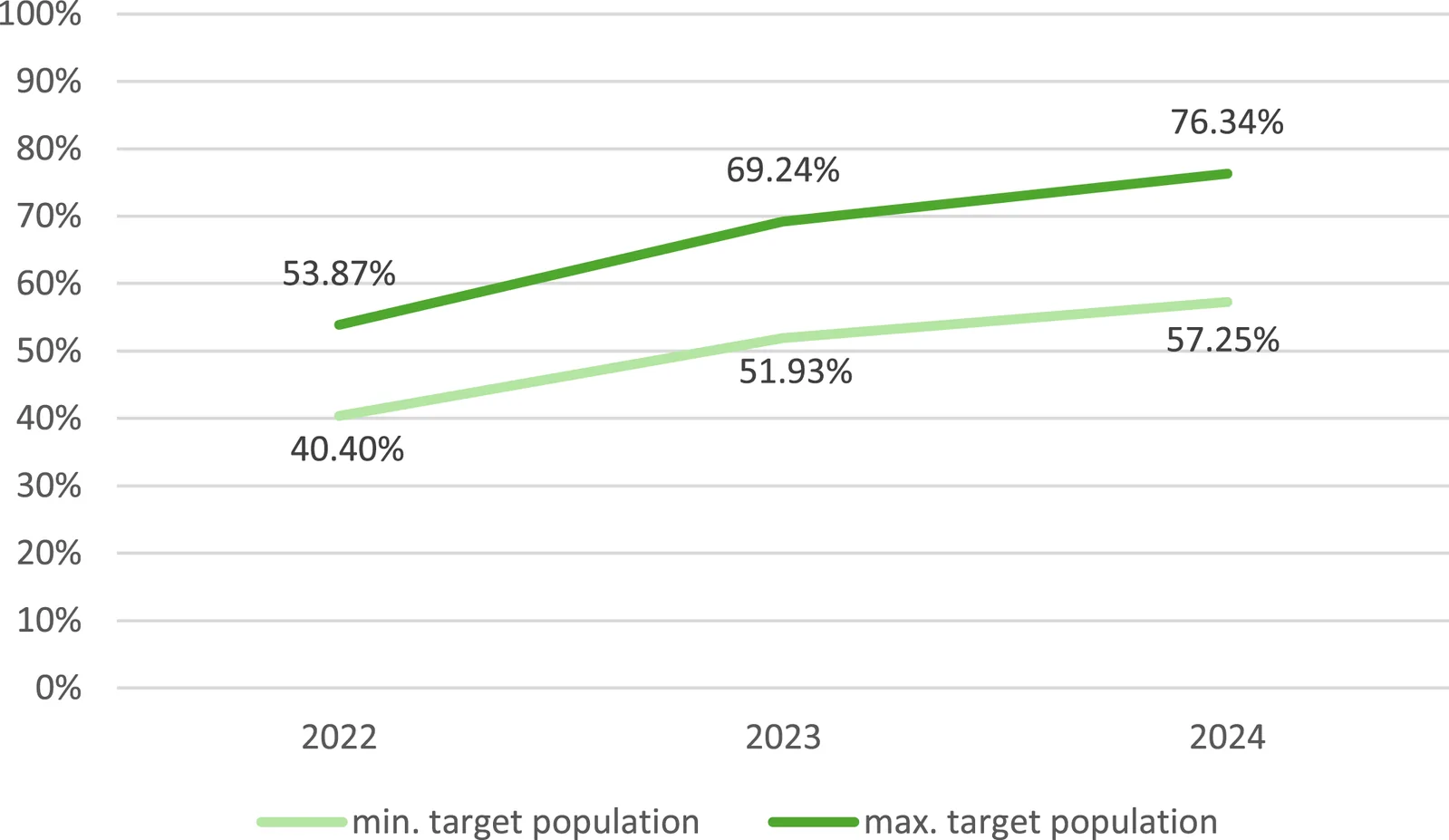
Development over time of the estimated transplant eligibility rate in DLBCL.

The sensitivity analysis conducted to verify the 2L base population (44% of patients require a 2L treatment) resulted in a sensitivity base population of 14,454 cases [18]. Dividing these figures by the number of transplant-eligible cases (*n* = 7,523) yielded a sensitivity target population of 52% of cases defined as intended transplant-eligible.

To assess the potential impact of Pola-BR-related coding bias, a third sensitivity analysis was conducted. Patients with a drug-specific OPS code for polatuzumab vedotin (*n* = 2,997) were excluded from the total number of identified cases in 2024 (*n* = 7,523), resulting in an adjusted case count of 4,526. Based on the estimated number of patients with relapsed or refractory DLBCL in 2024, the proportion of patients intended transplant-eligible ranges from 34% (sensitivity target population_min_) to 46% (sensitivity target population_max_).

## Discussion

This analysis used German real-world inpatient billing data to approximate the proportion of transplant-eligible cases as opposed to transplant-ineligible cases in a 2L therapy setting in DLBCL, assuming that coding of salvage chemotherapy serves as a (technical) proxy for transplant-eligibility. The analysis was intended to evaluate the most recent data (2024, at date of analysis) and use less recent data (2022, 2023) to confirm findings. Results showed a constantly rising share over the years 2022–2024, instead of a consistent target population size (Fig. 3). In all observed years, the approximated proportion of transplant-eligible cases exceeded 50 percent, with an average of 58%, suggesting a systematically larger transplant-eligible group than previously assumed [11, 16].

The reason for the increase primarily originated in the increasing sum of cases that have been coded with the selected salvage chemotherapy codes, while the baseline DLBCL population and, therefore, the approximated 2L population size remained rather stable. This was verified by the sensitivity analysis based on health insurance claim data which yielded a maximum 2L population of 14,454 cases versus 13,140 in the main analysis. From a clinical and epidemiological perspective, there is no clear rationale to expect a substantial increase in the proportion of transplant-eligible patients over time. The observed trend may instead be attributed to shifts in clinical treatment patterns that have led to an increased usage of salvage chemotherapy. According to the input from a clinical expert, three developments in clinical practice may account for this trend. First, the recent integration of CAR T-cell therapy into clinical routine represents a possible key factor [21]. Similar to stem cell transplantation, CAR T-cell administration requires bridging chemotherapy as a preparatory regimen [7, 8]. Since a broader proportion of patients can be considered eligible for CAR T-cell therapy compared to stem cell transplantation, the overall use of salvage chemotherapy might have increased accordingly [22]. Another relevant development may be the more frequent use of R-GDP over R-ICE or R-DHAP due to better tolerability [23]. Hence, salvage regimes could be used also for transplant-ineligible patients or even in palliative intent [8]. Third, there has been a continuous rise, particularly over the past 2 to 3 years, in the use of the polatuzumab vedotin in combination with bendamustin and rituximab (Pola-BR), which is not associated with stem cell transplantation as the Pola-BR regime is used for transplant-ineligible patients in 2L setting or later treatment lines [6, 19]. The sensitivity analysis, in which polatuzumab vedotin cases were excluded, estimated a slightly lower proportion of the transplant-eligible population. However, the actual clinical reason for the use of polatuzumab vedotin cannot be definitively validated based on the aggregated underlying billing data. For example, it could also be used in bridging intent for patients eligible for CAR-T cell therapy.

These clinical developments may translate into increased coding of salvage chemotherapy, as the codes used to identify bridging therapy before CAR T-cell therapy and ASCT salvage chemotherapy are largely indistinguishable. In addition, Pola-BR is often coded similarly to R-GDP, one of the regimens that were subject to this analysis, leading to an increase in selected codes [20]. However, it cannot be determined if this led to an increase in overall chemotherapy coding rather than solely a redistribution among chemotherapy regimens during the study period. An explanation solely based on changes in coding guidelines can largely be ruled out following a review of German inpatient coding instructions for the respective years [20, 24, 25]. Taken together, these mentioned clinical developments may be the key drivers of the observed temporal coding trend.

To date, the literature contains very few analyses that attempt to estimate the proportion of transplant-eligible versus transplant-ineligible patients. In the context of German health technology assessment processes, the analysis of Friedberg et al. [11] assuming a 50-50 distribution is primarily referenced. Since transplant eligibility is not defined by strict clinical parameters or standardized criteria but is instead determined on a case-by-case basis by the treating physician, a prospective approach to this research question is restricted [9]. For this reason, real-world data represent a valuable source for investigating this issue. The use of inpatient billing data as performed within this analysis offers an innovative approach that provides a relevant contribution to address this evidence gap.

Nonetheless, the following limitations lead to a certain degree of uncertainty regarding the presented results: first, this analysis exclusively considers inpatient hospital cases, disregarding any outpatient cases, as no centralized database of corresponding billing data is available. Although salvage chemotherapies are more likely to be applied in an inpatient setting, a minor underestimation of cases cannot be ruled out. Second, the estimated population of 2L cases could not be narrowed down to exclude cases beyond the second therapy line, which may lead to an overestimation of cases. Third, as Pola-BR is coded similarly to R-GDP and is not associated with ASCT, the reported proportion of transplant-eligible cases may be overestimated. Furthermore, this analysis focuses on patients intended for HD-ASCT, rather than those who actually clinically responded to salvage chemotherapy and received HD-ASCT, as such information is not recorded at the required clinical patient-level detail in the aggregated hospital billing data used in the analysis.

## Conclusion

In conclusion, this analysis presents a new approach to approximate the distribution of transplant-eligible versus transplant-ineligible DLBCL patients intended for HD-ASCT in a 2L treatment setting. Findings reveal deviations from the commonly assumed equal distribution between transplant-eligible and transplant-ineligible DLBCL patients. Although considering methodological limitations listed above, the data suggest a trend toward a rather higher share of transplant-eligible cases.

## Statement of Ethics

This analysis exclusively used publicly available, retrospective and aggregated hospital inpatient billing data provided by the German Institute of Hospital Remuneration (InEK). Therefore, no statement of ethics should be required.

## Conflict of Interest Statement

A-C Froitzheim, MS Kurte, T Poos and F Kron are employees of the VITIS GmbH, which was sponsored by Gilead Sciences GmbH in connection with the development of this manuscript. U Holtick has served on advisory boards (not related to this study) for BMS, Johnson & Johnson, Kite Pharma (a Gilead company), and Roche and received honoraria (unrelated to this study) for talks from AbbVie, BeOne, BMS, Johnson & Johnson, Kite Pharma (a Gilead company), Novartis and Miltenyi Biotec.

## Funding Sources

This study was funded by Gilead Sciences GmbH. The funder had no role in the design, data collection, data analysis, and reporting of this study.

## Author Contributions

A-C Froitzheim and MS Kurte: conceptualization, methodology, validation, investigation, and writing – review and editing. U Holtick: validation and writing – review and editing. T Poos: conceptualization, methodology, formal analysis, investigation, writing – original draft, review, and editing, visualization, and project administration. F Kron: conceptualization, supervision, project administration, and funding acquisition.

## Data Availability

Main data source: hospital billing data set for the years 2022–2024 provided by the German Institute of Hospital Remuneration (IneK), available at https://datenbrowser.inek.org/

## References

[1] Anderson JR , Armitage JO, Weisenburger DD. Epidemiology of the non-hodgkin’s lymphomas: distributions of the major subtypes differ by geographic locations. Non-hodgkin’s lymphoma classification project. Ann Oncol. 1998;9(7):717–20. 10.1023/a:1008265532487 9739436

[2] Nastoupil LJ , Bartlett NL. Navigating the evolving treatment landscape of diffuse large B-Cell lymphoma. J Clin Oncol. 2023;41(4):903–13. 10.1200/JCO.22.01848 36508700

[3] Habermann TM , Weller EA, Morrison VA, Gascoyne RD, Cassileth PA, Cohn JB, . Rituximab-CHOP versus CHOP alone or with maintenance rituximab in older patients with diffuse large B-Cell lymphoma. J Clin Oncol. 2006;24(19):3121–7. 10.1200/JCO.2005.05.1003 16754935

[4] Rovira J , Valera A, Colomo L, Setoain X, Rodríguez S, Martínez-Trillos A, . Prognosis of patients with diffuse large B cell lymphoma not reaching complete response or relapsing after frontline chemotherapy or immunochemotherapy. Ann Hematol. 2015;94(5):803–12. 10.1007/s00277-014-2271-1 PMC4374121 25501975

[5] Tilly H , Morschhauser F, Sehn LH, Friedberg JW, Trněný M, Sharman JP, . Polatuzumab vedotin in previously untreated diffuse large B-Cell lymphoma. N Engl J Med. 2022;386(4):351–63. 10.1056/NEJMoa2115304 PMC11702892 34904799

[6] European Medicines Agency . Polivy: EPAR-product information. 2025. [cited 2025 Nov 17]. Available from: https://www.ema.europa.eu/en/documents/product-information/polivy-epar-product-information_en.pdf

[7] Diagnostik AWMF . Therapie und Nachsorge für erwachsene Patient*innen mit einem diffusen großzelligen B-Zell-Lymphom und verwandten Entitäten. AWMF-Registernummer: 018/038OL; 2022. Available from: https://www.leitlinienprogrammonkologie.de/leitlinien/dlbcl/

[8] Lenz G , Chapuy B, Glaß B, Keil F, Klapper W, Nickelsen M, . [Internet]. Diffuses großzelliges B-Zell-Lymphom. 2024 [cited 2026 June 09]. Available from: https://www.onkopedia.com/de/onkopedia/guidelines/diffuses-grosszelliges-b-zell-lymphom

[9] Bachanova V , Perales M-A, Abramson JS. Modern management of relapsed and refractory aggressive B-cell lymphoma: a perspective on the current treatment landscape and patient selection for CAR T-cell therapy. Blood Rev. 2020;40:100640. 10.1016/j.blre.2019.100640 31780119

[10] Lu T , Zhang J, Xu-Monette ZY, Young KH. The progress of novel strategies on immune-based therapy in relapsed or refractory diffuse large B-cell lymphoma. Exp Hematol Oncol. 2023;12(1):72. 10.1186/s40164-023-00432-z PMC10424456 37580826

[11] Friedberg JW . Relapsed/refractory diffuse large B-Cell lymphoma. Hematology. 2011;2011(1):498–505. 10.1182/asheducation-2011.1.498 22160081

[12] Bundesausschuss G . Beschluss zu Glofitamab (Neues Anwendungsgebiet: diffuses großzelliges B-Zell-Lymphom, rezidiviert oder refraktär, Kombination mit Gemcitabin und Oxaliplatin, autologe Stammzelltransplantation nicht geeignet). 2025. [cited 2026 June 9]. Available from: https://www.g-ba.de/downloads/91-1385-1220/2026-01-13_Geltende-Fassung_Glofitamab_D-1206.pdf

[13] Bundesausschuss G . Beschluss zu Axicabtagen-Ciloleucel (Neubewertung nach Fristablauf: Diffus großzelliges B-Zell-Lymphom, hochmalignes B-Zell-Lymphom, nach 1 Vortherapie, Rezidiv innerhalb von 12 Monaten oder refraktär); 2024. [cited 2026 June 09]. Available from: https://www.g-ba.de/downloads/91-1385-1097/2024-12-19_Geltende%20Fassung_Axicabtagen_Ciloleucel_D-1078_D-890.pdf

[14] Kamdar M , Solomon SR, Arnason J, Johnston PB, Glass B, Bachanova V, . Lisocabtagene maraleucel versus standard of care with salvage chemotherapy followed by autologous stem cell transplantation as second-line treatment in patients with relapsed or refractory large B-cell lymphoma (TRANSFORM): results from an interim analysis of an open-label, randomised, phase 3 trial. Lancet. 2022;399(10343):2294–308. 10.1016/S0140-6736(22)00662-6 35717989

[15] Locke FL , Miklos DB, Jacobson CA, Perales M-A, Kersten M-J, Oluwole OO, . Axicabtagene ciloleucel as second-line therapy for large B-Cell lymphoma. N Engl J Med. 2022;386(7):640–54. 10.1056/NEJMoa2116133 34891224

[16] Gisselbrecht C , Schmitz N, Mounier N, Singh Gill D, Linch DC, Trneny M, . Rituximab maintenance therapy after autologous stem-cell transplantation in patients with relapsed CD20^+^ diffuse large B-Cell lymphoma: final analysis of the collaborative trial in relapsed aggressive lymphoma. J Clin Oncol. 2012;30(36):4462–9. 10.1200/JCO.2012.41.9416 PMC3646314 23091101

[17] Institut für das Entgeltsystem im Krankenhaus (InEK) [Internet] . InEK DataBrowser; 2025. [cited 2025 Nov 17]. Available from: https://datenbrowser.inek.org/

[18] Borchmann P , Heger J-M, Mahlich J, Papadimitrious MS, Riou S, Werner B. Survival outcomes of patients newly diagnosed with diffuse large B-cell lymphoma: real-world evidence from a German claims database. J Cancer Res Clin Oncol. 2023;149(10):7091–101. 10.1007/s00432-023-04660-y PMC11797273 36871091

[19] Sehn LH , Herrera AF, Flowers CR, Kamdar MK, McMillan A, Hertzberg M, . Polatuzumab vedotin in relapsed or refractory diffuse large B-Cell lymphoma. J Clin Oncol. 2020;38(2):155–65. 10.1200/JCO.19.00172 PMC7032881 31693429

[20] Thalheimer M . Kodierleitfaden für die Hämatologie/Onkologie 2024. medhochzwei Verlag; 2024.

[21] Hentrich M . Therapie maligner lymphome. Dtsch Z Onkol. 2022;54(02):60–7.

[22] Di Rocco A , Cuneo A, Di Rocco A, Merli F, De Luca G, Petrucci L, . Relapsed/Refractory diffuse large B-cell lymphoma patients. A multicenter retrospective analysis of eligibility criteria for car-T cell therapy. Leuk Lymphoma. 2021;62(4):828–36. 10.1080/10428194.2020.1849676 33274677

[23] Gong IY , Aminilari M, Landego I, Hueniken K, Zhou Q, Kuruvilla J, . Comparative effectiveness of salvage chemotherapy regimens and chimeric antigen T-cell receptor therapies in relapsed and refractory diffuse large B cell lymphoma: a network meta-analysis of clinical trials. Leuk Lymphoma. 2023;64(10):1643–54. 10.1080/10428194.2023.2234528 37548344

[24] Thalheimer M . Kodierleitfaden für die Hämatologie/Onkologie 2022. medhochzwei Verlag; 2022.

[25] Thalheimer M . Kodierleitfaden für die Hämatologie/Onkologie 2023. medhochzwei Verlag; 2023.

